# Recovery of pulmonary functions, exercise capacity, and quality of life after pulmonary rehabilitation in survivors of ARDS due to severe influenza A (H1N1) pneumonitis

**DOI:** 10.1111/irv.12566

**Published:** 2018-06-12

**Authors:** Meng‐Jer Hsieh, Wei‐Chun Lee, Hsiu‐Ying Cho, Meng‐Fang Wu, Han‐Chung Hu, Kuo‐Chin Kao, Ning‐Hung Chen, Ying‐Huang Tsai, Chung‐Chi Huang

**Affiliations:** ^1^ Department of Pulmonary and Critical Care Medicine Chia‐Yi Chang‐Gung Memorial Hospital Chang‐Gung Medical Foundation Chiayi Taiwan; ^2^ Department of Respiratory Therapy School of Medicine Chang‐Gung University Taoyuan Taiwan; ^3^ Department of Respiratory Therapy Lin‐Kou Chang‐Gung Memorial Hospital Chang‐Gung Medical Foundation Taoyaun Taiwan; ^4^ Department of Pulmonary and Critical Care Medicine Lin‐Kou Chang‐Gung Memorial Hospital Chang‐Gung Medical Foundation Taoyaun Taiwan

**Keywords:** acute respiratory distress syndrome, exercise capacity, influenza A H1N1, pulmonary function tests, quality of life

## Abstract

**Background:**

Acute respiratory distress syndrome (ARDS) due to severe influenza A H1N1 pneumonitis would result in impaired pulmonary functions and health‐related quality of life (HRQoL) after hospital discharge.

**Objectives:**

The recovery of pulmonary functions, exercise capacity, and HRQoL in the survivors of ARDS due to 2009 pandemic influenza A H1N1 pneumonitis (H1N1‐ARDS) was evaluated in a tertiary teaching hospital in northern Taiwan between May 2010 and June 2011.

**Patients and Methods:**

Data of spirometry, total lung capacity (TLC), diffusing capacity of carbon monoxide (DL_CO_), and 6‐minute walk distance (6MWD) in the patients survived from H1N1‐ARDS were collected 1, 3, and 6 months post‐hospital discharge. HRQoL was evaluated with St. George respiratory questionnaire (SGRQ).

**Results:**

Nine survivors of H1N1‐ARDS in the study period were included. All these patients received 2 months’ pulmonary rehabilitation program. Pulmonary functions and exercise capacity included TLC, forced vital capacity (FVC), forced expiratory volume in the first second (FEV_1_), DL_CO_, and 6MWD improved from 1 to 3 months post‐hospital discharge. Only TLC had further significant improvement from 3 to 6 months. HRQoL represented as the total score of SGRQ had no significant improvement in the first 3 months but improved significantly from 3 to 6 months post‐discharge.

**Conclusion:**

The impaired pulmonary functions and exercise capacity in the survivors of H1N1‐ARDS improved soon at 3 months after hospital discharge. Their quality of life had keeping improved at 6 months even though there was no further improvement of their pulmonary functions and exercise capacity.

## 1 INTRODUCTION

In March 2009, cases of flu‐like illness in Mexico caused by a novel H1N1 influenza virus were reported and this novel virus spread rapidly across the globe within weeks. It was estimated that approximately 23 000 persons had been infected in Mexico by late April, giving an estimated case fatality rate of 0.4%.^1^ More than one million people became ill with novel H1N1 influenza between April and June 2009 in the United States.^2^ During the period of pandemic novel influenza A H1N1 infection between July 2009 and June 2010, the collaborated laboratories of Taiwan Center for Disease Control (Taiwan CDC) received 16 372 samples for influenza testing. Of those, 3310 were positive for H1N1 influenza virus, accounting for 92% of the 3606 virus‐positive samples.^3^ There were 1809 complicated cases due to novel A H1N1 influenza infection during 2009‐2010 in Taiwan.^4^ Severe H1N1 pneumonitis would result in acute respiratory distress syndrome (ARDS) and multi‐organ failure, and associated with prolonged use of mechanical ventilation or even extracorporeal membrane oxygenation (ECMO).^5^ The mortality associated with severe H1N1 infections was reported as high as 20%‐40%.^6^ In a study of 320 pandemic influenza pneumonia in Japan, 43 (13.4%) had received invasive mechanical ventilation and 7.8% died of severe pneumonia.^7^

Pulmonary function impairment and reduced exercise capacity had been found in about half of the survivors of ARDS. One year after hospital discharge for ARDS, up to 80% of the patients demonstrated reduced diffusing capacity, one‐fifth of them had airflow obstruction, and one‐fifth had chest restriction.^8^ Besides the impairment of pulmonary function, the survivors of ARDS also had reduced health‐related quality of life.

The aim of this study was to assess the recovery from ARDS due to severe influenza A (H1N1) pneumonitis (H1N1‐ARDS). Pulmonary functions, exercise capacity, and health‐related quality of life in the survivors of H1N1‐ARDS after hospital discharge in a tertiary teaching hospital in northern Taiwan were analyzed.

## 2 SUBJECTS AND METHODS

### 2.1 Patients

Patients aged greater than 18 years with ARDS due to severe influenza A (H1N1) pneumonitis admitted to medical intensive care unit (ICU) at Linkou Chang‐Gung Memorial Hospital, Taiwan, between May 2010 and June 2011 and survived to be discharged from the hospital were eligible for analysis in this study. The influenza infection was confirmed either by real‐time RT‐PCR assay or influenza virus rapid test performed on the nasopharyngeal swab or specimens aspirated from the endotracheal tubes. Specimens were collected at the time of admission. Patients fulfilled with all of the listed criteria of the Berlin definition were diagnosed as ARDS^9^: (i) new or worsening symptoms during 1 week, (ii) bilateral opacities consistent with pulmonary edema be present on a chest radiograph or computed tomographic (CT) scan, (iii) the ratio of arterial oxygen tension to fraction of inspired oxygen less then 300 mm Hg with positive end‐expiratory pressure of 5 cm H_2_O or more, (iv) no clinical evidence of congestive heart failure or fluid overload. Patients with H1N1‐ARDS were transferred to the ward when they were hemodynamically stable and were liberated from mechanical ventilation. Those willing to participate pulmonary rehabilitation program received a course of up to 2 months’ exercise training, simplified strength training, and breathing exercise. They had regular follow‐up of pulmonary functions, exercise capacity, and St. George’s Respiratory Questionnaire (SGRQ) evaluation at 1, 3, and 6 months after hospital discharge. All data were retrieved from the hospital records. Those without pulmonary function tests, six‐minute walk test, or did not receive pulmonary rehabilitation were excluded for analysis. This study was approved by the Institutional Review Board of Chang‐Gung Medical Foundation, and patient consent was waived.

### 2.2 Pulmonary function tests

Pulmonary function testing (PFT) was performed using the MasterLab system (MasterLab; Jaegger, Germany) and was performed by technicians in the pulmonary function laboratory. All tests were proceeded according to American Thoracic Society guidelines.^10^ The expiratory volume in the first second (FEV_1_) and vital capacity (FVC) during forced expiration were recorded. Static lung volumes were measured using the plethysmography method, and diffusing capacity of the lung for carbon monoxide (DLco) was measured using the single breath‐hold method.^11^

### 2.3 Six‐minute walk test

Six‐min walk test (6MWT) was performed by respiratory therapists in the pulmonary rehabilitation center according to American Thoracic Society guidelines.^12^ The 6MWT was performed 1, 3, and 6 months after discharge from hospital. The distances the patients walked quickly in a period of 6 minutes (6MWD) were recorded.

### 2.4 Health‐related Quality‐of‐Life questionnaire

SGRQ was used to evaluate quality of life. The SGRQ results are grouped into 3 domains (symptoms, activity, and impacts) and a total score. A 4‐point change in SGRQ has been determined to be clinically significant difference.^13^ The SGRQ was also performed 1, 3, and 6 months after discharge from the hospital by respiratory therapists in the pulmonary rehabilitation center.

### 2.5 Statistical analysis

The measurements of pulmonary function testing, 6MWD, and scores of SGRQ at different time points were compared with Wilcoxon signed rank sum test. The statistical analyses were performed using the SPSS (SPSS for Windows, SPSS Inc., Chicago, IL, USA), and the significance level (α) was set at 0.05, and *P* < .05 was considered statistically significant.

## 3 RESULTS

### 3.1 General characteristics

Nine patients with confirmed ARDS due to severe influenza A (H1N1) pneumonitis admitted to medical ICU at Linkou Chang Gung Memorial Hospital, Taiwan, between May 2010 and June 2011 were recruited. The baseline characteristics of these patients are listed in Table 1. The mean age was 45.11 ± 5.48 years, and all were males. Two of these patients received ECMO support for 11 and 12 days. The mean mechanical ventilation use and ICU stay of these patients were 14.88 ± 2.38 days and 16.89 ± 2.51 days, respectively. The mean hospital stay was 33.55 ± 4.23 days. Most of the patients had no major comorbidity, except one had diabetes mellitus and the other two had hypertension. All these patients received 2 months’ pulmonary rehabilitation program including endurance exercise training with cycle ergometer, breathing exercise, and simplified weight training three times a week during hospitalization and after discharge from the hospital.

**Table 1:** Demographic data of survivors with ARDS due to severe influenza A (H1N1) pneumonitis

Cases	Gender	Age(mean ± SD)	BMI(Kg/M^2^)	ECMOdays	MV days(mean ± SD)	ICU days(mean ± SD)	Hospital days(mean ± SD)	Comorbidities
		45.11 ± 5.48	23.62 ± 4.76		14.88 ± 2.38	16.89 ± 2.51	33.56 ± 4.23	
1	Male	29	22.49	11	23	24	32	None
2	Male	28	28.73	‐	a	6	16	None
3	Male	47	20.07	‐	9	19	37	None
4	Male	35	21.47	‐	8	9	14	None
5	Male	40	28.09	‐	13	14	29	None
6	Male	54	24.21	‐	11	12	34	Hypertension
7	Male	37	31.16	12	27	29	43	None
8	Male	80	18.29	‐	13	23	45	Hypertension
9	Male	56	18.07	‐	15	16	52	Diabetes mellitus

a High flow oxygen mask, FiO2 = 80%.

### 3.2 Pulmonary function tests

**Figure 1:**
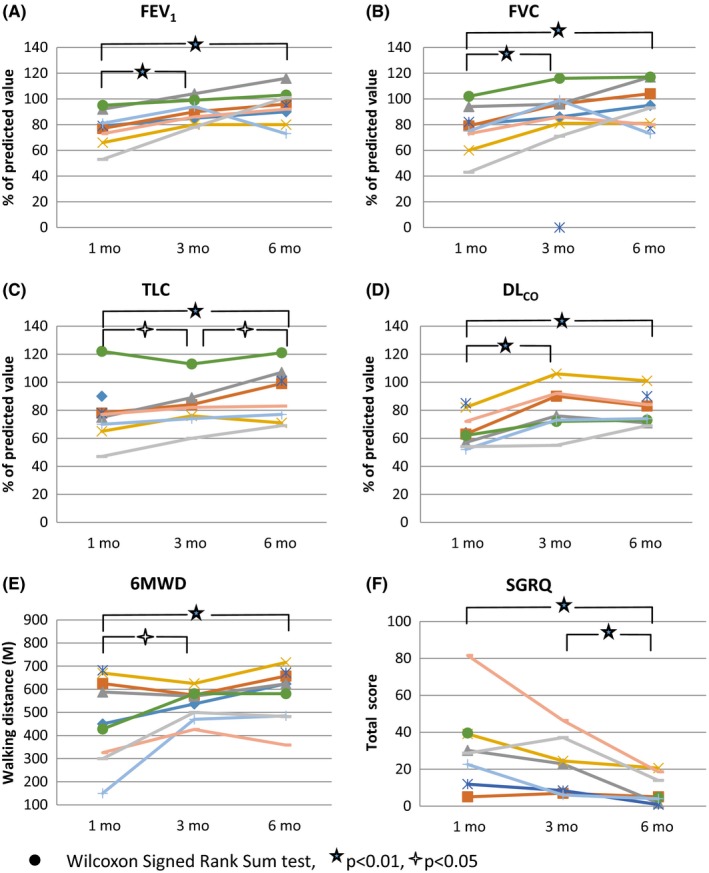
Measurements of FEV_1_, FVC, TLC, DL_CO_, 6MWD, and SGRQ in survivors with ARDS due to severe influenza A (H1N1) pneumonitis at 1, 3, and 6 mo after discharge from the hospital. ●Wilcoxon signed rank sum test, 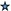 *P* < .01, 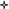 *P* < .05

According to the diagnostic criteria of American Thoracic Society guideline,^10^ six (66.7%) of 9 patients had mildly to moderately decreased FEV_1_ and 4 (44.4%) of 9 patients presented with mildly to moderately reduced FVC, and one (11.1%) had severely reduced FVC 1 month after discharge from the hospital (Figure 1A,B). At sixth months, all 6 patients with decreased FEV_1_ at baseline and 4 of the 5 patients with decreased FVC at baseline had their FEV_1_ or FVC returned to normal. Six (66.7%) of 9 patients had reduced total lung capacity (TLC) at 1 month and 4 of them (44.4%) remained with reduced TLC at 6 months (Figure 1C). Besides, seven (77.8%) of 9 patients showed reduced DL_CO_ at 1 month and four (44.4%) of them still presented with mild diffusing impairment at 6 months. Compared with the values at 1 month, FEV_1_, FVC, TLC, and DL_CO_ improved significantly 3 months after hospital discharge. Nevertheless, compared with the data at 3 months, there was no further significant improvement of lung function measurements except TLC at 6 months (Figure 1D).

### 3.3 Six‐minute walk test

Our patients covered a median 6MWD of 486.6 m (150‐682 m), 551 m (470‐625 m), and 604.8 m (482‐716 m) at 1, 3, and 6 months after hospital discharge, represented 28%‐100%, 76%‐93%, and 65%‐107% of the predicted value.^14^ (Figure 1E) The pattern of changes in 6MWD during the 6 months’ follow‐up is similar as in FEV_1_, FVC, and DL_CO_. 6MWD improved at 3 months, and no further significant improvement was identified at 6 months.

### 3.4 Health‐related Quality of Life (HRQoL)

Eight of nine patients had completed their SGRQ. All but one H1N1‐ARDS survivor were associated with worse pulmonary disease‐specific HRQoL in all 3 domains at baseline. Compared with baseline SGRQ total score, all but one of our patients had 4 or more points decrease at 6 months after discharge, with median values decreased from 29.4 to 4.8 during the 6‐month follow‐up. The only one without significant decrease in total score of SGRQ had a low score of 5 at 1 month and remained 5 at 6 months. The improvement of total score of SGRQ did not reach to a statistical difference in the initial 3 months (*P* = .064), but there was a significant improvement from 3 to 6 months after discharge from the hospital (Figure 1F).

## 4 DISCUSSIONS

Although most patients suffered from influenza A (H1N1) infection had a mild, self‐limiting illness of the upper respiratory tract, a certain percentage of patients developed progressive severe pneumonia and ARDS.^15^, ^16^, ^17^ ARDS with severe hypoxemia and requiring mechanical ventilation or even extracorporeal membrane oxygenation (ECMO) support^18^ is associated with high mortality and morbidity despite advanced treatment strategies. Prone positioning and even ECMO were more frequently applied, and ICU stay was significantly longer in patients with H1N1‐ARDS, although there was no difference in ICU survival compared with non‐H1N1‐ARDS patients.^19^ These patients experienced not only physical morbidities but also mental and psychological stress that resulted in significantly impairment of health‐related quality of life.^20^, ^21^ Therefore, the contemporary objective of intensive care in these patients was not only to assure their survival, but also to attain an ideal outcome of quality of life.

**Figure 2:**
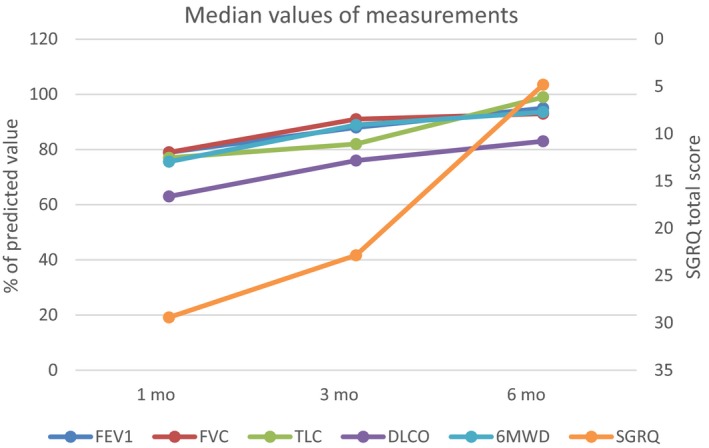
Median values of FEV_1_, FVC, TLC, DL_CO_, 6MWD, and total score of SGRQ at 1, 3, and 6 mo after discharge from the hospital

In this observational study, we followed the survivors of ARDS resulted from severe influenza A (H1N1) pneumonitis up to 6 months after hospital discharge to evaluate their functional recovery and improvement of health‐related quality of life. Pulmonary function tests including FEV_1_, FVC, TLC, DL_CO_, and exercise capacity presented as 6MWD improved significantly in the first 3 months but with no further significant improvement from 3 to 6 months after discharge. TLC improved continuously during the 6‐month follow‐up. Their quality of life, represented as total score of SGRQ, had no statistically significant improvement (*P* = .064) in the first 3 months, but the mean difference of total score (‐9.6) had exceeded minimally clinical improvement difference of 4. The total score of SGRQ decreased significantly from 3 to 6 months after discharge, although most of pulmonary function measurements and exercise capacity did not increase further in this period (Figure 2).

At the end of 6 months’ follow‐up, all of our patients with abnormal baseline FEV_1_ and 80% of those with abnormal baseline FVC had their FEV_1_ or FVC returned to normal. The exercise capacity presented as 6MWD, all returned to normal at 6 months. The recovery of diffusing capacity of the lung was not as good as FEV_1_, FVC, or 6MWD; there were 4 (44.4%) of 9 patients still had impaired DL_CO_ (<80% of predicted value) at 6 months post‐hospital discharge.

There are few articles reported the recovery of pulmonary functions, exercise capacity, and quality of life in the patients survived from ARDS due to severe influenza A H1N1 pneumonitis.^20^, ^22^, ^23^ Quispe‐Laime and his coworkers reported 11 patients with ARDS due to influenza A H1N1 infection, all patients had normal spirometry and DL_CO_ at 6 months after discharge.^22^ Toufen et al reported none of three patients had abnormal DL_CO_ at 6 months.^23^ Luyt and his coworkers demonstrated that pulmonary function test results were nearly normal at one‐year post‐ICU discharge and only 5.4% of patients had mild obstruction. Twenty‐five of 37 patients (67.6%) in that study still had impaired diffusing capacity at 1 year.^20^ These reports had no demonstrable data earlier than 6 months after discharge. With data collected at 1, 3, and 6 months after hospital discharge, most patients in our study had well‐recovered pulmonary functions at 3 months. Furthermore, their TLC had significant continuous improvement till the end of 6 months’ follow‐up. Our results demonstrated the fast recovery of pulmonary functions and exercise capacity in the early stage after hospital discharge in the patients survived from H1N1‐ARDS.

In survivors of ARDS not caused by influenza A H1N1, Orme and his coworkers found that approximately 80% of ARDS patients had reduced diffusing capacity, 20% had airway obstruction, and 20% had chest restriction at 1 year after recovery.^8^ Another study demonstrated 67% of the patients with ARDS had some alteration of pulmonary function at 6 months after diagnosis. The PFT alteration was characterized by a restrictive pattern in 58% of cases, an obstructive pattern in 6%, and a mixed pattern in 3%.^21^ Ong et al also presented some survivors from severe acute respiratory syndrome who had mild impairment of FVC and DL_CO_ during 3 months’ follow‐up.^24^ Our patients, with ARDS due to influenza A H1N1, seemed to have a better pulmonary function recovery at 3 and 6 months after discharge. None of our patients had abnormal FEV_1_ at 6 months, and 22.2% had mildly reduced FVC, 44.4% had slightly lower TLC, and 44.4% had mildly impaired diffusing capacity. The inconsistent results in the recovery of pulmonary functions after discharge between H1N1 and non‐H1N1 ARDS probably resulted from the heterogeneous patient characteristics and different severities in lung inflammation and damage.

There were two articles evaluated exercise capacity in patients survived from H1N1‐ARDS. Toufen et al reported two of three patients had low 6MWD at 6 months.^23^ Luyt et al demonstrated the mean values of maximal oxygen consumption in patients with or without extracorporeal lung assist (ECLA) at 1 year post‐ICU were 80.5% and 89%, respectively.^20^ Unlike the results of previous reports with certain percentages of H1N1‐ARDS patients having impaired exercise capacity at 6 months or 1 year after discharge, all of our patients had their 6MWD returned to normal (>80% of predicted value) at 6 months post‐discharge. The recovery of exercise in our patient might partly benefit from the pulmonary rehabilitation program.

Reduced pulmonary function was significantly correlated with impaired quality of life and exercise capacity. The pulmonary function abnormalities correlated with decreased health‐related quality of life for domains reflecting physical function in survivors of ARDS.^8^ Schelling and colleagues demonstrated that patients with more abnormality on pulmonary function tests had the lowest health‐related quality of life.^25^ In patients with H1N1‐ARDS, Quispe‐Laimea et al found there was no correlation between lung diffusion and quality of life at 6 months.^22^ In our patients, the quality of life measured by SGRQ improved but not reached to statistical significance in the first 3 months despite significant improvement in pulmonary function tests and 6MWD. The total score of SGRQ decreased significantly from 3 to 6 months, although the measurements of pulmonary functions and 6MWD had no significant increase during this period. Our findings indicated that there would be a further improvement of quality of life even after the improvement of pulmonary functions or exercise capacity reached to plateau levels.

All of our patients received 2 months’ pulmonary rehabilitation program with exercise training, simplified strength training, and breathing exercise. Prior study showed ARDS patients have both initial and persistent debilitating limitations in physical function. The declines in physical function may even persist at 8 years.^26^ Several studies had reported pulmonary rehabilitation can help to improve exercise capacity, sleep quality, depression, and health‐related quality of life. With the limited number of patients included in this study, we did not compare the outcomes between H1N1‐ARDS patients with and without pulmonary rehabilitation. All of the patients in our study received similar pulmonary rehabilitation program because we thought that pulmonary rehabilitation could be beneficial to the recovery of physical status in these severely ill patients. Therefore, this could partly explain why our patients had more significant improvement after survived form H1N1‐ARDS. However, further studies are needed to identify the optimal therapeutic interventions in a timely and targeted manner.

There are several limitations in this study. First, the case number was small because of not all of the patients survived from ARDS due to influenza A H1N1 pneumonitis had regular follow‐up of their pulmonary function tests and exercise tests. Second, a non‐influenza control group was lacking and the difference in the recovery from ARDS resulted from severe H1N1 infection or other causes is not clarified. Third, we did not compare the outcomes between H1N1‐ARDS patients with and without pulmonary rehabilitation, and the effects of pulmonary rehabilitation to the recovery of H1N1‐ARDS patients still remained unclear. Besides, we did not assess other variables, such as heart and neuromotor function, which might affect their pulmonary functions or health‐related quality of life.

## 5 CONCLUSION

Although our study series is small, the results suggest that patients survived from ARDS secondary to influenza A H1N1 pneumonitis had impaired pulmonary functions, exercise capacity, and quality of life after hospital discharge. The impaired pulmonary functions and exercise capacity improved soon at 3 months after hospital discharge, and their quality of life had keeping significant recovery up to 6 months.

## References

[1] Fraser C , Donnelly CA , Cauchemez S , et al. Pandemic potential of a strain of influenza A (H1N1): early findings. Science. 2009;324:1557‐1561. 19433588 10.1126/science.1176062 PMC3735127

[2] Center for Disease Control and Prevention, USA . 2009 H1N1 Early Outbreak and Disease Characteristics. 2009 [Available from: https://www.cdc.gov/h1n1flu/surveillanceqa.htm Accessed March 03, 2018.

[3] Center for Disease Control, Taiwan, ROC . Influenza surveillance weekly report, week 26, 2010 2010 [Available from: http://flu.cdc.gov.tw/public/Data/0761673071.pdf accessed June 20, 2012.

[4] Center for Disease Control, Taiwan, ROC . Statistics of Communicable Diseases and Surveillance Report 2011. 2011 [Available from: http://www.cdc.gov.tw/uploads/files/201301/472cb943-8774-4015-bcd6-ca27e85524e0.pdf accessed June 27, 2017.

[5] Australia and New Zealand Extracorporeal Membrane Oxygenation Influenza Investigators , Davies A , Jones D , et al. Extracorporeal membrane oxygenation for 2009 Influenza A(H1N1) acute respiratory distress syndrome. JAMA 2009;302:1888‐1895. 19822628 10.1001/jama.2009.1535

[6] Kumar A . Pandemic H1N1 influenza. J Thorac Dis. 2011;3:262‐270. 22263101 10.3978/j.issn.2072-1439.2011.08.05 PMC3256529

[7] Fujikura Y , Kawano S , Kouzaki Y , et al. Mortality and severity evaluation by routine pneumonia prediction models among Japanese patients with 2009 pandemic influenza A (H1N1) pneumonia. Respir Investig. 2014;52:280‐287. 10.1016/j.resinv.2014.04.003 25169843

[8] Orme J , Romney JS , Hopkins RO , et al. Pulmonary function and health‐related quality of life in survivors of acute respiratory distress syndrome. Am J Respir Crit Care Med. 2003;167:690‐694. 12493646 10.1164/rccm.200206-542OC

[9] The ARDS Definition Task Force . Acute respiratory distress syndrome: the berlin definition. JAMA. 2012;307:2526‐2533. 22797452 10.1001/jama.2012.5669

[10] Miller MR , Hankinson J , Brusasco V , et al. Standardisation of spirometry. Eur Respir J. 2005;26:319‐338. 16055882 10.1183/09031936.05.00034805

[11] Macintyre N , Crapo RO , Viegi G , et al. Standardisation of the single‐breath determination of carbon monoxide uptake in the lung. Eur Respir J. 2005;26:720‐735. 16204605 10.1183/09031936.05.00034905

[12] American Thoracic Society . ATS statement: guidelines for the six‐minute walk test. Am J Respir Crit Care Med. 2002;166:111‐117. 12091180 10.1164/ajrccm.166.1.at1102

[13] Jones PW . St. George’s respiratory questionnaire: MCID. COPD. 2005;2:75‐79. 17136966 10.1081/copd-200050513

[14] Enright PL , Sherrill DL . Reference equations for the six‐minute walk in healthy adults. Am J Respir Crit Care Med. 1998;158(5 Pt 1):1384‐1387. 9817683 10.1164/ajrccm.158.5.9710086

[15] Dominguez‐Cherit G , Lapinsky SE , Macias AE , et al. Critically Ill patients with 2009 influenza A(H1N1) in Mexico. JAMA. 2009;302:1880‐1887. 19822626 10.1001/jama.2009.1536

[16] ANZIC Influenza Investigators , Webb SA , Pettila V , et al. Critical care services and 2009 H1N1 influenza in Australia and New Zealand. N Engl J Med. 2009;361:1925‐1934. 19815860 10.1056/NEJMoa0908481

[17] Kumar AZRPR , et al. Critically ill patients with 2009 influenza A(H1N1) infection in canada. JAMA. 2009;302:1872‐1879. 19822627 10.1001/jama.2009.1496

[18] Abrams D , Brodie D . Novel uses of extracorporeal membrane oxygenation in adults. Clin Chest Med. 2015;36:373‐384. 26304275 10.1016/j.ccm.2015.05.014

[19] Topfer L , Menk M , Weber‐Carstens S , et al. Influenza A (H1N1) vs non‐H1N1 ARDS: analysis of clinical course. J Crit Care. 2014;29:340‐346. 24508203 10.1016/j.jcrc.2013.12.013

[20] Luyt CE , Combes A , Becquemin MH , et al. Long‐term outcomes of pandemic 2009 influenza A(H1N1)‐associated severe ARDS. Chest. 2012;142:583‐592. 22948576 10.1378/chest.11-2196

[21] Masclans JR , Roca O , Munoz X , et al. Quality of life, pulmonary function, and tomographic scan abnormalities after ARDS. Chest. 2011;139:1340‐1346. 21330382 10.1378/chest.10-2438

[22] Quispe‐Laime AM , Fiore C , Gonzalez‐Ros MN , et al. Lung diffusion capacity and quality of life 6 months after discharge from the ICU among survivors of acute respiratory distress syndrome due to influenza A H1N1. Med Intensiva. 2012;36:15‐23. 22118978 10.1016/j.medin.2011.09.007

[23] Toufen C Jr , Costa EL , Hirota AS , et al. Follow‐up after acute respiratory distress syndrome caused by influenza a (H1N1) virus infection. Clinics (Sao Paulo). 2011;66:933‐937. 21808854 10.1590/S1807-59322011000600002 PMC3129942

[24] Ong K‐C , Ng AW‐K , Lee LS‐U , et al. 1‐year pulmonary function and health status in survivors of severe acute respiratory syndrome. Chest. 2005;128:1393‐1400. 16162734 10.1378/chest.128.3.1393 PMC7094739

[25] Schelling G , Stoll C , Vogelmeier C , et al. Pulmonary function and health‐related quality of life in a sample of long‐term survivors of the acute respiratory distress syndrome. Intensive Care Med. 2000;26:1304‐1311. 11089757 10.1007/s001340051342

[26] Iwashyna TJ , Ely EW , Smith DM , Langa KM . Long‐term cognitive impairment and functional disability among survivors of severe sepsis. JAMA. 2010;304:1787‐1794. 20978258 10.1001/jama.2010.1553 PMC3345288

